# Low-Fat *Tenebrio molitor* Meal as a Component in the Broiler Diet: Growth Performance and Carcass Composition

**DOI:** 10.3390/insects15120979

**Published:** 2024-12-11

**Authors:** Evgeni Petkov, Teodora Popova, Krasimir Dimov, Desislava Vlahova-Vangelova, Desislav Balev, Nikolay Kolev, Stefan Dragoev

**Affiliations:** 1 Agricultural Academy, Institute of Animal Science-Kostinbrod, Pochivka Str, 2232 Kostinbrod, Bulgaria; e_petkov@ias.bg; 2 Agricultural Academy, Institute of Cryobiology and Food Technologies, 53 Cherni Vrah Blvd, 1407 Sofia, Bulgaria; krasimir.dimov@ikht.bg; 3 Department of Meat and Fish Technology, University of Food Technologies, 26 Maritsa Blvd, 4002 Plovdiv, Bulgaria; d_vangelova@uft-plovdiv.bg (D.V.-V.); d_balev@uft-plovdiv.bg (D.B.); n_kolev@uft-plovdiv.bg (N.K.); s_dragoev@uft-plovdiv.bg (S.D.); 4 Bulgarian Academy of Sciences, 1, 15 Noemvri Str, 1014 Sofia, Bulgaria

**Keywords:** *Tenebrio molitor*, low-fat insect meal, broilers, growth performance, carcass quality

## Abstract

*Tenebrio molitor* is considered a sustainable protein source for diets in poultry. It has been investigated in regard to the performance, health status, and product quality in different poultry species. This study was designed to evaluate the effect of low-fat *Tenebrio molitor* meal (LFTM) included in the broilers’ diet on the growth performance and carcass composition of the birds. The trial was conducted at the Institute of Animal Science-Kostinbrod, Bulgaria. A total of 120 male one-day-old Ross 308 broilers were allocated to 5 groups. After an adaptation period of 14 days, the birds were fed a basal diet (C) and diets including LFTM in amounts of 2.5% (T2.5), 5% (T5), 7.5% (T7.5), and 10% (T10). The growth performance of the birds was measured during the trial. At 35 days of age, 8 birds per group were slaughtered, and their carcass quality was evaluated. We found a linear response of BWG and FCR towards the inclusion of *Tenebrio molitor* meal (TM) in the diet. For the whole period (14–35 d), the BWG decreased (*p* = 0.0002), while the values of FCR increased (*p* = 0.0001) with the increase in the levels of the insect meal. The doses of 7.5% and 10% of the insect meal were associated with lower carcass weight and also decreased the yield of the breast. The results of the study indicated that the incorporation of LFTM, especially in amounts of 7.5–10%, could not be appropriate for broilers due to adverse effects on the growth performance and carcass composition.

## Simple Summary

*Tenebrio molitor*, or yellow mealworm, has demonstrated strong potential as a sustainable protein source in the diet of poultry. This study aims to investigate the effect of different doses of low-fat *Tenebrio molitor* meal included in the diet of Ross 308 broilers on their growth performance and carcass composition. We found that the inclusion of low-fat *Tenebrio molitor* meal at an amount of 10% in the diet affected negatively the body weight gain (BWG) and the feed conversion ratio (FCR) of the birds. The doses of 7.5% and 10% of the insect meal were associated with lower carcass weight, a lower percentage of the breast, and a higher percentage of the back in the broilers.

## 1. Introduction

Insects appear to be a promising alternative protein source showing high potential to be included as a sustainable component in food and feed. Their use for this purpose has been the subject of numerous studies in recent years due to the rising concerns for future global food insecurity due to the rapidly increasing population [1]. Although considerable interest has been attracted recently in the possibilities to incorporate insect protein in human diets in meat and cereal products [2,3,4,5,6,7], still insects are primarily included in the animals and poultry diets to replace soybean or fishmeal [8,9]. Worldwide, chicken meat production has steadily increased in the last decade from 83,267 t in 2012 to 103,549 in 2023 [10]. This is driven by the increased meat consumption, the high awareness of the health benefits this kind of meat proposes [11,12], the lack of the religious constraints [13], and also the greater affordability of the chicken meat compared to the red meats [14]. The major cost in raising the broilers is feeding, as the latter might have significant environmental impact [15,16]. To reduce the latter, it is necessary to find available, efficient, and sustainable alternative feed sources, insects being a possible solution. In addition to the protein, insects are a valuable source of polyunsaturated fatty acids, minerals, and vitamins [17,18]; require fewer resources to farm [19]; and also they are natural food for the birds, thus enhancing their welfare [20,21].

In 2017, with Regulation (EU) 2017/893 of 24 May [22], the use of the black soldier fly (*Hermetia illucens*), the common housefly (*Musca domestica*), the yellow mealworm (*Tenebrio molitor*), the lesser mealworm (*Alphitobius diaperinus*), the house cricket (*Acheta domesticus*), the banded cricket (*Gryllodes sigillatus*), and the field cricket (*Gryllus assimilis*) was allowed as feed in aquaculture. Later in 2021, their use was extended to poultry and pig feeds with Regulation (EU) 2021/1372 of 17 August [23]. Mostly, the insects are incorporated in the poultry diet as meals. The latter are low-cost and environmentally friendly products that meet poultry requirements in terms of nutritional value, essential amino acid composition, nutrient digestibility, and feed acceptance [24]. Furthermore, they contain antimicrobial peptides and bioactive molecules that can substantially improve the health status of the poultry [25,26,27,28].

Yellow mealworm (*Tenebrio molitor*) contains significant amounts of protein rich in essential amino acids [20,24]. According to Elahi et al. [29], the essential amino acid index is higher than that of soybean and comparable to fishmeal. *Tenebrio molitor* meal has been investigated in regard to the growth performance [30,31,32,33,34,35,36], haematological profile [29,31,34,36], carcass traits [29,30,32,34], meat quality [29,37], intestinal morphology [38,39], immunological traits [33] in broilers. The results of the studies so far have been contradictory, and the effect on the growth performance, carcass, and meat quality was found to be dependent on the dose. The meals derived from *Tenebrio molitor* incorporated in the poultry diets contained significant amounts of fat (approx. 30%). Insect meals can also be defatted. Decreasing the fat leads to a substantial increase in the crude protein content and better resistance to degradation when compared to full-fat insect meals [40]. The fat content of the defatted *Tenebrio molitor* larvae meal ranges within 5.7–8.1%, while the protein content is 63–71.3% [41]. To our knowledge, the studies on the inclusion of *Tenebrio molitor* defatted or low-fat meals have been carried out mainly with different fish species [42,43,44]. The incorporation of such ingredients in poultry diets is rather scarce and studied in quails [45]. Hence, the aim of the study was to evaluate the influence of the low-fat *Tenebrio molitor* meal included in different doses in the diet of broilers on the growth performance and carcass composition.

## 2. Materials and Methods

### 2.1. Ethics Statement

The experimental protocol used in this study, including the animal management and housing, was designed in compliance with the guidelines of the European and Bulgarian legislation regarding the protection of animals used for experimental and other scientific purposes [46]. The protocol was based on the permit for use of animals in experiments no. 227 of the Bulgarian Food Safety Agency (statement no. 193 of the Bulgarian Animal Ethics Committee, prot.no.18/02.07.2020).

### 2.2. Experimental Birds and Housing

The experiment was carried out in the Institute of Animal Science-Kostinbrod, Bulgaria, with a total of 120 male Ross 308 broilers. The one-day-old chickens were supplied by Hris Ltd., Pazardzik, Bulgaria, and were randomly allocated to 5 groups. Each group included 4 replicate cages with 6 birds per cage. The trial lasted 35 days and was carried out in appropriately designed premises in the experimental poultry farm of the Institute. The environmental conditions were set following the recommendations of Aviagen [47] as presented in Table 1:

The premises were equipped with artificial fluorescent light and forced ventilation. Heating was achieved through local brooders on each cage. The health of the birds was monitored twice daily. At hatching, the chickens were vaccinated against Newcastle disease, Marek disease, and infectious bronchitis. At 9 and 21 days of age, the broilers were vaccinated against infectious bursal disease.

### 2.3. Tenebrio Molitor Larvae

For the purposes of the trial, LFTM was supplied by VBF Pro Ltd., Petrich, Bulgaria, and its chemical composition is presented in Table 2. The insects were fed pig starter feed mixed with wheat bran (1:4 to 1:6 according to the development of the larvae). Additionally, the insects were supplemented with potatoes, carrots, or water 2–3 times per week. The larvae were reared at 25 °C and RH 55–60% until 10–12 weeks of age. Then they were sacrificed and dried in a conventional oven at 180 °C for 7 min. The dried larvae were further processed to meal for the purposes of the experiment.

### 2.4. Experimental Diets

The feeding of the broilers was in two phases, as during the first two weeks of the trial (1–14 d), all the groups were fed the same starter diet adjusted to the standards for Ross broilers. For the grower period, four experimental diets, including LFTM, were formulated as presented in Table 3. During the trial period, the birds received feed and water ad libitum.

### 2.5. Growth Performance

The mortality of the birds was controlled daily. The live weight (LW) and the feed intake (FI) were recorded weekly. These parameters were further used to calculate BWG and FCR.

### 2.6. Slaughtering Procedures and Carcass Analysis

At the end of the trial, two birds per replicate were selected from each group based on the average live weight. After stunning, decapitation, and bleeding, the carcasses were plucked, eviscerated, and their feet removed. Hot carcass weight was recorded, and dressing percentage was calculated as follows:

Dressing % = (HCW/LW) × 100, %, where:

HCW—hot carcass weight of the bird;

LW—live weight of the bird.

The carcasses were then stored at 4 °C for 24 h and weighed again. Further, the abdominal fat was removed from the carcasses, and they were separated into breast, thigh, back, and wings. The carcass parts were weighed and calculated as a percentage of the cold carcass.

### 2.7. Statistical Evaluation

Statistical processing using the JMP v. 7 software package [48]. The normality of distribution for the data were checked by the Shapiro–Wilk test. The cage was the experimental unit for the growth performance, while the individual chicken was used for the carcass parameters. Data were subjected to one-way ANOVA. Polynomial contrasts were used to test the linear and quadratic effects of the increasing levels of LFTM in the diet of the birds. The differences between the means in the groups were tested using the Tukey HSD test (*p* < 0.05). Data were presented as mean and standard error of the mean (SEM).

## 3. Results

### 3.1. Growth Performance

No significant differences were detected in the initial live weight between the groups (Table 4).

**Figure 1:**
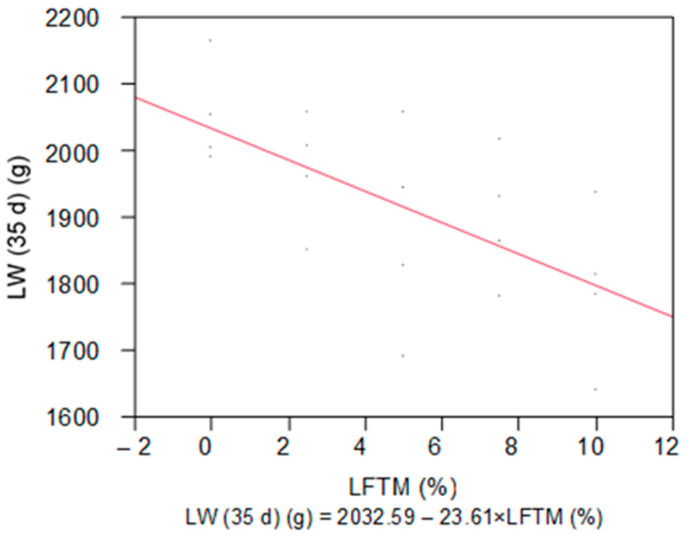
Linear response of the final LW to the dose of the LFTM in the diet of broilers.

During the first week of the adaptation period, the live weight of the birds did not differ between the groups; however, at the end of the period (14 d), the broilers from two of the groups tended to be heavier (*p* = 0.0960). The live weight of the broilers remained similar between groups in the first week of the feeding with LFTM. Afterwards there was a linear decrease in the values of the trait at 28 d (*p* = 0.0092) and 35 d (*p* = 0.0026). The linear response of the final LW to the dose of the LFTM is presented in Figure 1.

During the adaptation, there was no significant difference in the BWG of the broilers between groups; however, its values tended to be higher in T7.5 and T10 (*p* = 0.0936). After the start of the feeding with the insect meal, BWG responded linearly to the increase in its content in the diet and decreased for the period 15–21 d (*p* = 0.0433), 22–28 d (*p* < 0.0001), and 29–35 (*p* = 0.0262). The lowest BWG was recorded in T10 birds, while the control broilers had the highest values of this trait.

The values of AFI differed significantly between groups during the adaptation (*p* = 0.0150), with the highest value observed in T10. During the feeding, including LFTM, AFI did not differ between groups; however, a trend towards linear increase (*p* = 0.0811) was observed in the period 15–21 d in the groups consuming higher levels of the insect meal.

Despite the significant differences in the feed intake and the tendencies in BWG during the adaptation, they did not induce considerable differences in the FCR. However, significant linear increase was observed in FCR values during 15–21 d (*p* = 0.0028) and 22–28 d (*p* < 0.0001). During 29–35 d this trait also tended to increase linearly (*p* = 0.0507).

**Figure 2:**
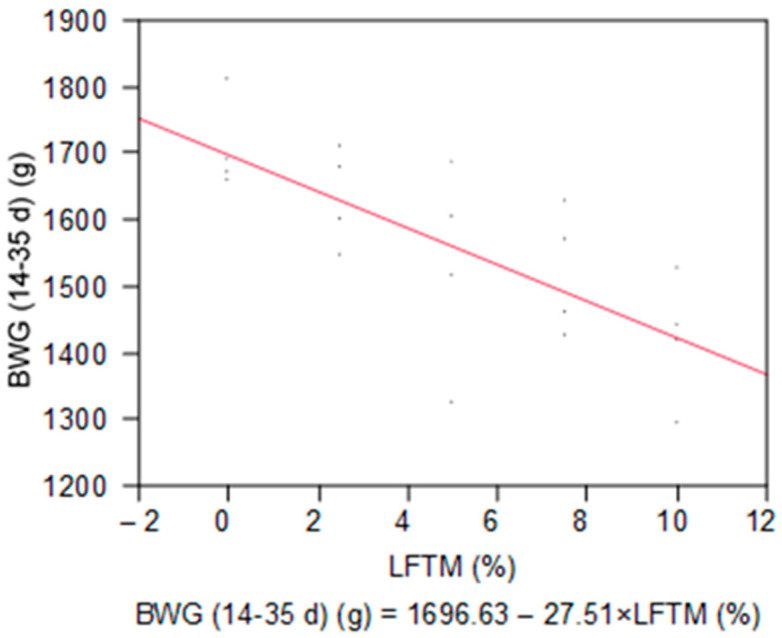
Linear response of BWG to the dose of the LFTM in the diet of broilers.

For the whole period of feeding LFTM, the feed intake remained unaffected in all the groups, while the BWG decreased significantly with increasing the levels of the insect meal (*p* = 0.0002) (Table 4, Figure 2).

**Figure 3:**
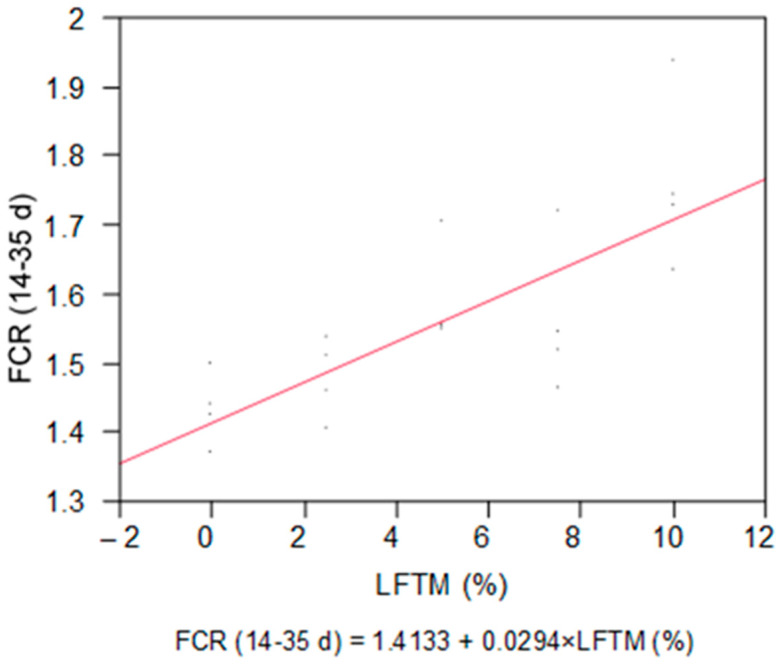
Linear response of FCR to the dose of the LFTM in the diet of broilers.

The minimal BWG was detected in the broilers from the T10 group. The diminishing of the BWG in the groups that consumed a higher percentage of insect meal led to increased values of the FCR for the whole period (*p* = 0.0001) (Table 4, Figure 3).

### 3.2. Carcass Analysis

The addition of LFTM in the diet of the broilers significantly affected the warm and cold carcass weight, as both decreased linearly (*p* < 0.0001), reaching minimal values at 10% of the meal in the diet (Table 5).

Linear response was observed in the percentage of breast (*p* < 0.0001) and thighs (*p* = 0.0235). The percentage of the breast decreased with increasing the percent of the added insect meal. The highest value of this parameter was measured in the control group and the lowest in the group supplemented with 7.5% and 10% LFTM (*p* < 0.05). The opposite pattern was observed in regard to the percentage of the thighs, where the values of this parameter were lowest in the control group. The back of the birds also responded linearly to the insect meal in the diet (*p* = 0.0002). The highest percentage of this carcass part was observed in the groups fed 7.5% and 10% LFTM, while the lowest value was recorded in the control group. In the neck we observed a quadratic response, but again the highest percent was found in the birds from the T7.5 and T10 groups.

## 4. Discussion

Although the inclusion of *Tenebrio molitor* in the diet of animals and poultry has been the subject of other studies, still the results for its potential are contradictory. During the whole experimental period, the LW of the birds did not differ significantly among groups; however, it tended to be lower in the birds from T7.5 and T10. In contrast to our study, Biasato et al. [38] showed a linear increase in the LW in broilers at 12 and 25 days of age with maximum values at 15% and 10% TM meal, respectively. However, at 53 days of age, the birds tended to decrease their weight when receiving the higher doses of the insect meal. Nieto et al. [49] investigated the effect of TM larvae meal as a total replacement of the soybean meal in the diet of slow-growing chickens. They observed a decrease in the LW and BWG in the indoor phase until 50 days of age of the birds, which is in agreement with our results. The lowest LW observed in the group fed 10% LFTM in our experiment corresponded to its lowest BWG for the whole trial period. The observed linear response of this trait to the incorporation of the insect meal in the diet showed its adverse effect on the BWG in the broilers. This, however, was not observed by Sedgh-Gooya et al. [34,39], who showed a positive effect of TM meal (2.5% and 5%) on the BWG in broilers in the starter phase. Hussain et al. [50] also reported a positive effect of the TM meal at lower doses (1–3 g/kg diet) on the BWG of the birds. Andrade et al. [35] reported higher BWG in broilers receiving 2% of the insect meal; however, no effect of the supplement in lower doses (0.5%) was detected. An earlier study of Ramos-Elorduy et al. [51] showed no significant effect of the inclusion of TM larvae meal (5% and 10%) in the diet of 7 week old Arbor Acres × Vantress broilers for a two-week trial on the weight gain. Such results were later confirmed by Bovera et al. [31] in broilers from 30 to 62 days of age fed 30% of the insect meal. Our results are consistent with those of Jiang et al. [36], who showed that the addition of high doses of TM meal in the diet of broilers can be detrimental to body weight and the average daily gain. The authors observed a negative effect on these traits at 7% and 9% of the insect meal in the diet. In line with our results, Sarica et al. [45] also found a significant decrease in the BWG of quails fed a diet with 75% and 100% replacement of the fish meal by defatted mealworm larvae meal. The authors also reported a significant decrease in the final LW of the birds.

The AFI in our experiment was not affected by the inclusion of the insect meal in the diet of the birds. This is consistent with other studies on the potential of *Tenebrio molitor* as a protein source in poultry diets, reporting no effect of the products derived from this insect on the feed intake of the birds [31,36,39]. Our results, however, contradict the results of Biasato et al. [38] for substantially increased feed intake in the broilers at 5%, 10%, and 15% TM meal. A study of Šťastník et al. [52] showed a decrease in the feed intake of broilers when receiving 2% and 5% TM larvae meal. A negative impact on the feed intake produced by *Tenebrio molitor* dried whole larvae at 5% in the diet of broilers was also reported by Vasilopoulos et al. [53]. However, according to this study, no effect on the feed intake was observed at 10% TM larvae in the diet. In an earlier study, Khan et al. [54] observed lower FI in broilers receiving TM meal in the diet (8.1%) compared to the birds fed a basal diet or such supplemented with maggot or silkworm meals. Bovera et al. [31] observed lower FI in broilers receiving a diet with TM meal as a full substitute for soybean protein.

For the whole trial, the FCR was within the range of 1.43–1.75. Substantial differences between groups were observed during the separate periods, showing the negative effect of the high levels of LFTM (10%) on the FCR. This agrees with the results of Terera et al. [55], who reported a negative effect of TM larvae meal on the FCR at doses higher than 5%. The authors concluded that the high levels of meal derived from *Tenebrio molitor* have a detrimental effect on the performance indicators of broilers and can be added up to 5% in the diet. Impaired FCR was also reported by Biasato et al. [38], while Bovera et al. [31] showed improved FCR in the group fed TM meal. Doses higher than 1.6% were found to slightly impair the growth performance and linearly increase FCR in Japanese quails [56]. The increased values of FCR in the groups fed high levels of LFTM in this experiment corresponded to the lower BWG of these birds. It might be suggested that such an effect on the growth performance is due to the high chitin level of *Tenebrio molitor*; however, this warrants further research. Earlier hypotheses stated that insect chitin might decrease the nutrient digestibility, particularly that of the protein [57], possibly through its binding to digestive enzymes or its embedding in a matrix of proteins, lipids, and minerals that could decrease the accessibility of the digestive enzymes to these components [58]. On the other hand, a study of Tabata et al. [59] demonstrated that in chickens, acidic chitinase can function as a digestive enzyme that breaks down chitin-containing organisms in the chicken gastrointestinal tract. This enzyme degraded the shells of the mealworm larvae in the presence of digestive proteases to produce (GlcNAc)2 and a few GIcNAc fragments in the stomach and the intestines. However, the effect of the products produced after chitin degradation on the development and health of the chickens has to be clarified. Nutritional factors are known to play a key role in modulating immunity and host-parasite relationships, so further research is needed in order to evaluate how the addition of LFTM, due to its peculiar nutritional profile, interacts with gut health and immunity [60] and hence affects the digestibility of the nutrients.

The effect of the different doses of the LFTM on the carcass weights corresponds to those in the LW, and the highest dose produced carcasses with lower weight. The dressing percentage varied within the range of 75.69–78.47%. The lower carcass weight of the birds fed the maximal dose (10% LFTM) was associated with a lower percentage of the breast and a higher percentage of the back and neck. In contrast to our results, Bovera et al. [32] did not observe an effect of TM larvae meal on the carcass weight in broilers. Biasato et al. [61] also reported no negative effect on the carcass and its parts in free-range chickens fed TM meal. The study of Elahi et al. [29] showed no effect of insect meal in the diet of broilers at amounts of 2, 4, 8, and 10.48%. Šťastník et al. [52] observed no response in the carcass weight, breast, and thigh yield in broilers receiving 2% and 5% TM meal, which is in agreement with our results. Higher doses of defatted TM larvae meal (50, 75, 100%) replacing fish meal also negatively affected the carcass weight in Japanese quails [45]. Zadeh et al. [62] found that the inclusion of *Tenebrio molitor* in the diet of quails increased breast yield at 0.75%, 2.25%, and 3% and also increased leg yield at 1.5% and 3% in the diet.

The results from our experiment contradict most of the studies so far focused on the *Tenebrio molitor* as a possible alternative protein source in poultry diets. The main reason is the use of the low-fat meal in our study. Furthermore, the discrepancies that were demonstrated could be attributed to the nutritional profile of the larvae used, which is affected by the rearing substrate, and also to the different periods of bird feeding trials.

## 5. Conclusions

Feeding low-fat *Tenebrio molitor* meal for three weeks did not positively affect the growth performance of the broilers and their carcass composition. Although the live weight of the birds did not differ significantly among the groups, the body weight gain decreased linearly with increasing the percentage of the insect meal in the diet. Furthermore, the higher doses of the meal were associated with increased values of feed conversion ratio and also produced carcasses with lower weight and decreased percentage of the breast. Based on the results of the study, the incorporation of low-fat *Tenebrio molitor* meal, especially in amounts of 7.5–10%, could not be appropriate for broilers. Further research is needed to build strategies for the inclusion of low-fat mealworm meal in the poultry diets, possibly combined with oils, so that the growth performance and carcass composition of the birds are not compromised.

## Figures and Tables

**Table 1:** Environmental conditions of the broilers.

Age	Light Period	Dark Period	Light Intensity	Temperature	RH
1 day old	24 h	0 h	20–60 lux	32 °C	50–60%
2–7 days	23 h (1 × 23 h light spot)	1 h (1 × 1 h dark spot)	20–60 lux	28–32 °C	40–50%
8–35 days	12 h (4 × 3 h light spot)	12 h (4 × 3 h dark spot)	5–10 lux	22–28 °C	40–50%

**Table 2:** Chemical composition of the LFTM incorporated in the broilers’ diet.

Moisture, %	Crude Protein, %	Crude Fat, %	Crude Fiber, %	Crude Ash, %	Nitrogen Free Extracts, %	Ca, %	P, %
11.74	62.8	4.02	9.46	7.47	4.51	0.082	1.536

**Table 3:** Composition of the broilers’ diet used in the trial.

Component, %	C	T2.5	T5	T7.5	T10
Wheat	36.51	38.46	40.03	41.83	43.90
Soybean meal	35.80	32.00	28.50	24.90	20.90
Maize	20.00	20.00	20.00	20.00	20.00
Sunmeal oil	3.40	2.95	2.60	2.15	1.70
Monocalcium phosphate	1.11	1.05	1.00	0.92	0.85
Limestone	0.90	0.95	1.00	1.05	1.10
Mollasses	0.50	0.50	0.50	0.50	0.50
DL-Methionine	0.34	0.33	0.31	0.30	0.28
Sodium bicarbonate	0.20	0.19	0.18	0.13	0.12
L-Lysine	0.31	0.23	0.13	0.03	0.01
L-Treonine	0.12	0.08	0.05	0.01	0.01
Sacox	0.05	0.05	0.05	0.05	0.05
Choline Chloride	0.05	0.05	0.05	0.05	0.05
Salt	0.20	0.15	0.09	0.07	0.02
Paradigmox	0.01	0.01	0.01	0.01	0.01
Vitamin mineral premix ^1^	0.50	0.50	0.50	0.50	0.50
Tenebio molitor meal	0.00	2.50	5.00	7.50	10.00
Chemical composition					
Crude protein, %	23.05	22.99	23.01	23.02	22.95
Crude fat, %	5.02	4.65	4.38	4.00	3.63
Crude fiber, %	2.57	2.72	2.87	3.03	3.18
Crude ash, %	5.25	5.16	5.08	4.99	4.90
Metabolizable energy, kcal/kg	3001.32	3000.18	3002.17	3000.34	3001.03

^1^ The vitamin-mineral premix provided the following per kg of diet: I, 1.56 mg; Fe, 50 mg; Cu, 12.50 mg; Zn, 100 mg; Mn, 125 mg; Se, 0.31 mg; vit. A, 12,500 IU; vit. D3, 5000 IU; vit. E, 100 mg; vit. K3, 3.41 mg; vit. B1, 2.50 mg; vit. B2, 7.50 mg; calcium D panthotenate, 15 mg; vit. B6, 4.38 mg, vit. B12, 25 mcg, folic acid, 1.88 mg; biotin, 125 mcg; vit. B3, 62.50 mg; 6-phytase, 500 FTU; BHA, 0.21 mg; BHT, 4.32 mg; propyl gallate, 2.17 mg; endo 1,4 beta-xylanase, 1100 VU, endo1,3-beta glucanase, 1500 VU.

**Table 4:** Growth performance of the broilers as affected by the incorporation of LFTM in the diet.

Trait	Age	C	T2.5	T5	T7.5	T10	SEM ^1^	*p*-Value		
								ANOVA	Linear	Quadratic
LW ^2^,g	1 d	40.66	40.90	42.08	41.36	40.97	1.132	0.4570	0.5586	0.1463
	7 d	136.90	132.00	127.96	136.00	131.80	6.721	0.3707	0.5803	0.3140
	14 d	344.78	335.74	346.45	377.86	372.50	23.940	0.0960	0.0189	0.5460
	21 d	753.66	772.42	723.62	778.48	749.46	40.990	0.3866	0.9728	0.9226
	28 d	1391.07	1384.38	1284.23	1316.22	1264.58	71.019	0.0765	0.0092	0.7558
	35 d	2048.90	1965.08	1875.09	1894.73	1789.00	112.887	0.0524	0.0026	0.7520
BWG ^3^,g	1–14 d 15–21 d	304.12 408.88 ^ab^	294.85 436.68 ^a^	304.38 377.17 ^b^	336.49 400.63 ^ab^	331.53 376.96 ^b^	23.776 24.880	0.0936 0.0210	- 0.0433	- 0.7245
	22–28 d	637.42 ^a^	611.95 ^ab^	560.61 ^bc^	537.74 ^bc^	515.13 ^c^	34.330	0.0007	<0.0001	0.5853
	29–35 d	657.83	580.71	590.86	578.51	524.42	73.698	0.2095	0.0262	0.8637
Overall	15–35 d	1704.12 ^a^	1629.34 ^ab^	1528.64 ^ab^	1516.88 ^ab^	1416.50 ^b^	102.149	0.0117	0.0002	0.8392
AFI ^4^, g/bird	1–14 d 15–21 d	387.31 ^ab^ 673.14	369.97 ^b^ 718.06	376.80 ^ab^ 690.52	410.34 ^ab^ 717.15	416.20 ^a^ 737.21	19.430 45.011	0.0150 0.3281	- 0.0811	- 0.9583
	22–28 d	789.79	811.04	787.35	809.86	794.79	43.698	0.8978	0.8940	0.7424
	29–35 d	972.81	869.99	940.39	830.69	945.58	94.819	0.2364	0.5684	0.1886
Overall	15–35 d	2435.74	2399.09	2418.26	2357.70	2477.58	124.236	0.7299	0.8271	0.3150
FCR ^5^	1–14 d 15–21 d	1.27 1.64 ^b^	1.25 1.64 ^b^	1.24 1.83 ^ab^	1.22 1.79 ^ab^	1.25 1.96 ^a^	0.056 0.140	0.726 0.0344	- 0.0028	- 0.6336
	22–28 d	1.24 ^d^	1.33 ^cd^	1.40 ^bc^	1.51 ^ab^	1.48 ^a^	0.062	<0.0001	<0.0001	0.4933
	29–35 d	1.48 ^ab^	1.50 ^ab^	1.59 ^ab^	1.44 ^b^	1.80 ^a^	0.178	0.0397	0.0507	0.2215
Overall	15–35 d	1.43 ^b^	1.47 ^b^	1.58 ^ab^	1.55 ^b^	1.75 ^a^	0.090	0.0013	0.0001	0.3655

Values connected with different superscripts are significantly different (*p* < 0.05); ^1^ Standard error of mean; ^2^ Live weight; ^3^ Body weight gain; ^4^ Average feed intake; ^5^ Feed conversion ratio.

**Table 5:** Carcass composition of the broilers as affected by the incorporation of LFTM in the diet.

Trait	C	T2.5	T5	T7.5	T10	SEM ^1^	*p*-Value		
							ANOVA	Linear	Quadratic
**HCW ^2^**	1762.63 ^a^	1642.50 ^b^	1586.38 ^bc^	1516.63 ^c^	1485.00 ^c^	74.313	<0.0001	<0.0001	0.1146
**CCW ^3^**	1702.50 ^a^	1576.63 ^b^	1526.88 ^bc^	1467.00 ^bc^	1429.17 ^c^	75.888	<0.0001	<0.0001	0.1113
**Dressing %**	77.91	78.47	77.56	77.16	75.69	2.567	0.3726	0.0743	0.3188
**Breast, %**	40.91 ^a^	38.17 ^b^	37.64 ^b^	34.60 ^c^	36.39 ^c^	2.298	0.0003	<0.0001	0.0764
**Thighs, %**	30.92 ^b^	31.78 ^ab^	32.12 ^ab^	33.51 ^a^	31.99 ^ab^	1.340	0.0246	0.0235	0.0931
**Back, %**	15.61 ^b^	17.15 ^bc^	17.47 ^bc^	18.77 ^a^	18.20 ^a^	1.394	0.0023	0.0002	0.1221
**Neck, %**	2.48 ^b^	2.50 ^b^	2.61 ^ab^	2.85 ^a^	2.87 ^a^	0.284	0.0349	0.6677	0.0019
**Wings, %**	10.08	10.40	10.16	10.27	10.55	0.447	0.3292	0.1543	0.7448

Values connected with different superscripts are significantly different (*p* < 0.05); ^1^ Standard error of mean; ^2^ Hot carcass weight; ^3^ Cold carcass weight.

## Data Availability

The data presented in this study are available upon request from the corresponding authors.
